# Mapping sponsorship portfolios in esports: implied strategic continuity and diversification in the league of legends pro league

**DOI:** 10.3389/fspor.2025.1601238

**Published:** 2025-10-02

**Authors:** Pengyu Zhu, Agudamu, Siyuan Zhao, Yang Zhang

**Affiliations:** ^1^ College of Physical Education, Hunan Normal University, Changsha, China; ^2^ Graduate School of Social Welfare, Sungkyunkwan University, Seoul, Republic of Korea; ^3^ Faculty of Humanities and Arts, Macau University of Science and Technology, Macao, Macao SAR, China; ^4^ Independent Person, Windermere, FL, United States

**Keywords:** advertising, marketing, sustainability, partnership, portfolio, volatility

## Abstract

**Objective:**

Although corporate sponsorship is the primary controllable revenue stream in professional esports, its strategic management—particularly regarding continuity and diversification—remains largely under-theorized. This gap is notable given the volatility and rapid commercialization of top-tier leagues. Focusing on China's League of Legends Pro League, this study examines how sponsorship portfolios are structured and sustained over time.

**Methods:**

Using 2021–2024 data from 17 clubs and the league, sponsorship continuity was quantified via Jaccard similarity coefficients and diversification via Blau indices across 25 MSCI industry categories.

**Results:**

Tencent-managed league-level portfolios were both stable and diversified, while most club portfolios were volatile and concentrated. Publicly held clubs achieved significantly greater diversification than private clubs, reflecting governance advantages. Club performance predicted greater subsequent diversification but not continuity. Positioning clubs within a continuity–diversification matrix revealed only a few in the “Strategic Resilience” quadrant, with many “At Risk.”

**Conclusion:**

These findings suggest that clubs should prioritize a core set of enduring sponsors while expanding into diverse categories to reduce revenue volatility, strengthen bargaining power, and enhance resilience to market shocks. The study provides a replicable framework for evaluating sponsorship continuity and diversification, supporting evidence-based decision-making in esports managerial practice.

## 1 Introduction

Corporate sponsorship has become a central driver of revenue in professional sports, yet its strategic management in esports remains under-theorized. In particular, the dual dimensions of continuity (retaining sponsorship partners over time) and diversification (spreading sponsorships across industries) are rarely examined systematically. This is a significant gap because esports organizations operate in highly volatile commercial environments, where sponsorship is often the most controllable revenue stream and the foundation for competitive sustainability.

The League of Legends Pro League (LPL) offers a valuable case for addressing this gap. As China's premier professional esports competition, the LPL operates under Tencent's oversight, combining centralized league-level sponsorship with decentralized club-level partnerships. The LPL's sponsorship ecosystem has faced mounting challenges—most recently, Riot Games' 2025 decision to permit betting companies as sponsors, raising ethical and reputational risks, especially among youth audiences. Such shifts underscore the need to understand how sponsorship portfolios are managed, and whether sustainable models can be achieved under economic, regulatory, and cultural pressures.

In light of these developments, this study investigates whether sustainable, long-term sponsorship management in esports is achievable under current conditions. By quantitatively assessing sponsorship portfolios within the LPL from 2021 to 2024, this research offers empirical evidence and new managerial insights into strategic resilience—and the lack thereof—in the world's most commercially successful esports league.

## 2 Literature review

Sponsorship has emerged as a foundational element in the commercial structure of professional sports. The helix sports marketing model conceptualizes this relationship as a triadic interaction among sponsors, media, and consumers, emphasizing mutual value creation through sustained engagement and brand exposure (1). While this model has matured in traditional sports, its adaptation to esports remains underdeveloped and unstable. Many esports organizations, despite attracting large audiences, lack diversified and dependable income streams. For instance, the NIP Group—the first publicly held esports club from China—reported losses of $6.3 million and $13.3 million in 2022 and 2023, respectively (2), highlighting the fragile financial foundations of esports operations.

Two dominant business models are used to conceptualize the esports club economy. The competition model includes revenues from sponsorships, prize winnings, athlete transfers, and league revenue sharing. However, this model alone is often insufficient for financial sustainability. For example, the League of Legends Champions Korea (LCK) clubs receive only approximately $600,000 per year from revenue sharing agreements—far below the operational needs of a top-tier esports organization (3). The brand operation model, in contrast, focuses on pan-entertainment activities (4) such as content production, influencer marketing, and live streaming. Chinese esports clubs are increasingly embracing this model by leveraging fan engagement and co-creating value with sponsors through localized activities, such as community events, amateur tournaments, and influencer-led product marketing.

Despite these innovations, empirical evidence across regions shows that most esports clubs operate at a loss (2, 5, 6). Ticket sales, merchandising, and in-stadium advertising remain underdeveloped. In this context, sponsorship stands out as the most immediate and controllable revenue stream. Clubs often rely on these deals not only to fund daily operations but also to attract elite talent and build their commercial identity. Yet, despite the critical importance of sponsorship, no academic study has examined how esports clubs manage sponsorship portfolios over time. This is a notable gap, especially given the global shifts in esports sponsorship practices. Although structural and financial conditions vary widely across regions—particularly between politically-linked ecosystems like China's LPL and market-driven leagues like the League Championship Series (LCS) in North America or LCK—the pressures to secure sustainable funding are shared across all top-tier esports leagues.

In seeking a theoretical foundation for effective sponsorship strategy, sports management literature offers important insights. In traditional sports, especially football, hierarchical sponsorship systems and long-standing partnerships are common. FIFA's structure includes tiered global sponsors, such as Adidas and Visa, which provide consistent financial support across tournament cycles. Similarly, clubs like Real Madrid adopt hybrid strategies: they maintain core partnerships with multinational corporations (e.g., Emirates) while engaging in regional deals that enable brand localization and flexibility (7). This model allows for both operational stability and strategic growth.

Empirical studies have further demonstrated that continuity in sponsorship—the retention of sponsor relationships across seasons—correlates with both financial performance and on-field success (8). This is partly due to the formation of a positive feedback loop: a consistent sponsor base supports planning and talent acquisition, which in turn improves performance and brand value. Moreover, diversified sponsorship portfolios protect clubs from reputational or geopolitical shocks. Recent examples include Bayern Munich's termination of its partnership with Qatar Airways under public pressure (9) and the global Boycott Puma campaign due to political affiliations (10). These cases show that overreliance on a single sponsor or industry can expose clubs to sudden and significant losses. Diversification also expands a club's network of stakeholders (11), creating more predictable and resilient revenue streams (12). This mirrors financial portfolio theory, where asset diversification reduces risk and increases return stability. Clubs that actively cultivate a mix of sponsors across sectors tend to perform better during crises and enjoy broader market visibility.

Despite the relevance of these insights, the esports field lacks empirical tools to measure sponsorship continuity and diversification systematically. Existing research remains qualitative or descriptive, often focused on branding, audience engagement, or the psychological appeal of esports. Little attention has been paid to how sponsorship portfolios evolve or how different managerial strategies affect the financial health of clubs and leagues. Moreover, no study to date has quantitatively assessed sponsorship structural similarity across seasons, or mapped portfolio diversity across sectors using standardized classification systems.

This study addresses that gap by introducing a quantitative framework to measure implied sponsorship strategies in esports, using the LPL as a case study. We operationalize continuity through the volatility of year-over-year Jaccard similarity coefficients in sponsor composition, and diversification through the Blau index across 25 industry groups from the MSCI classification system. In strategic terms, continuity captures a club's capability to retain and renew sponsor relationships across seasons, which lowers churn risk, preserves activation learning, and supports long-horizon collaboration. Diversification reflects the breadth of sponsor categories represented in the portfolio, which spreads revenue risk, reduces exposure to category-specific shocks, and signals wider brand reach. Considered jointly, high continuity and high diversification indicate strategic resilience because the portfolio is both stable and buffered against idiosyncratic shocks, whereas low values on both dimensions signal commercial fragility.

In doing so, this study contributes a novel empirical lens to the growing literature on esports management and offers actionable insights for club executives, league organizers, and potential sponsors navigating an increasingly competitive and uncertain sponsorship landscape in esports leagues worldwide.

## 3 Methods

### 3.1 Data

The LPL was founded in 2013, and the league statutes have undergone several revisions, leading to the implementation of a 17-club league format starting in 2020. The annual tournaments are split into spring and summer seasons, with each season further divided into a regular season and playoffs. Several sponsors reassessed their sponsorship choices in light of club performance, leading to periodic revisions in sponsorship throughout the year. Thus, this study analyzed the sponsorships of the regular season in spring for each year.

Due to a few ownership changes that occurred in 2021, the club names from 2022 onward were used retroactively for all 2021 clubs to ensure year-over-year consistency. The sponsorship landscape analyzed spans the 2021–2024 seasons. The league's and clubs' ownership and sponsorship details were acquired from publicly disclosed information and Tianyacha profiles. A complete list of sponsorship can be accessed at Figshare (DOI: 10.6084/m9.figshare.29420831.v1).

### 3.2 Analytic strategy

This study utilized R's igraph package (version 2.0.3), dplyr package (version 1.1.4), and Hmisc package (version 5.1-3) for data analysis. Quantifying the implied continuity strategy involved two steps. First, the Jaccard similarity coefficient was used to statistically map the year-over-year structural similarity of each club's sponsorship portfolio. The Jaccard coefficient is commonly applied in network analysis, management science, and strategic decision-making to evaluate compositional stability and relational proximity (13–15). Equation 1 defines the mathematical expression for the Jaccard similarity coefficient of sponsorship between year 202*x* and year 202*x* + 1.

(1) $$J(\bar{202x},\;\bar{202x+1})=\frac{\left|S202x\cap S202x+1\right|}{\left|S202x\cup S202x+1\right|}$$

The Jaccard similarity index ranges from zero (no shared sponsors across seasons) to one (identical sponsor lists). To measure a club's implied continuity strategy, we computed the standard deviation of its Jaccard similarity values from 2021 to 2024. This volatility index reflects the continuity or volatility of each club's sponsor structure. A lower index implies the presence of a coherent continuity strategy unless disrupted by deliberate or structural changes.

Sponsorship diversification was assessed by applying Blau's index across 25 industry groups defined by MSCI's 2023 Global Industry Classification Standard (16). Sponsors were assigned to one primary group based on dominant business activity; for instance, JD.com was categorized under “consumer discretionary distribution & retail,” while Samsung was grouped under “technology hardware & equipment.” Government-sponsored regional development zones such as Charming Longgang and Qujiang New District were categorized under “equity real estate investment trusts.” The computation is expressed in Equation 2 as follows:

(2) $$\mathrm{BI}it=1-\sum_{i=1}^{S}\;p_{it}^{2}$$

where BI*_it_* is the Blau index for league/club *i* in year *t*, *s* is the total number of categories a sponsor can belong to, and *p_it_* is the proportion of sponsors in league/club *i* that belong to category *s* at time *t*.

The Blau index measures the probability that two randomly selected sponsors come from different industry groups. Higher values reflect greater heterogeneity in the sponsorship portfolio, which is generally associated with resilience and strategic diversification in organizational theory (17).

To investigate the impact of corporate ownership on sponsorship strategy, we conducted Welch's *t*-test comparing the continuity and diversification metrics between publicly held and private clubs. Additionally, to facilitate the interpretation of the Continuity–Diversification relationship, we constructed a conceptual matrix in which each club was positioned according to its continuity score (calculated as one minus volatility index, such that higher values represent greater continuity in sponsorship relationships) and its Blau index. Four quadrants were defined using median splits of both metrics, calculated from the full dataset including the LPL benchmark point. Specifically, the horizontal threshold was set at the median continuity score and the vertical threshold at the median Blau index. This approach yielded four categories: Strategic Resilience (above-median Blau, above-median Continuity), defined by robust and adaptable sponsorship structures; Stable Niche (above-median Blau, below-median Continuity), characterized by a strong spread of sponsor categories but weaker long-term contract retention; Growth Potential (below-median Blau, above-median Continuity), featuring secure sponsorship ties but limited reach across categories; and, At Risk (below-median Blau, below-median Continuity), marked by low diversification and low continuity, marking them as more vulnerable to sponsorship loss and revenue volatility.

To test the robustness of the findings, we calculated two additional metrics. First, sponsorship continuity was also assessed using the *E* − *I* index (18), a network-theoretical metric defined in Equation 3.

(3) $$EI=\frac{E-I}{E+I}$$

where *E* (external ties) is the count of intergroup ties (i.e., identical sponsors of consecutive years), and *I* (internal ties) is the count of intragroup ties (i.e., disjoint sponsors of consecutive years).

The E-I index is bounded between negative one and one. A score of negative one indicates that the LPL or a club had no identical sponsors in consecutive years, representing homophily in social network analysis. Conversely, a score of one indicates that the LPL or a club had the same sponsors every year, representing heterophily in social network analysis. Second, Shannon's entropy was computed as a complementary diversification index (Equation 4), which has been used evaluating managerial approaches (19).

(4) $$H_{it}=-\sum_{i=1}^{S}\;p_{it}\ln\;(p_{it})$$

where *H_it_* is Shannon's entropy for league/club *i* in year *t*, *s* is the total number of categories a sponsor can belong to, and *p_it_* is the proportion of sponsors in league/club *i* that belong to category *s* at time *t*. Shannon's entropy, in practical terms, ranges from zero to infinity. A higher value suggests a greater level of diversification in the sponsored industry groups within this context. Next, we assessed the convergent validity between the Blau index and Shannon's entropy, as well as between the Jaccard similarity coefficient and the E-I index, by calculating Pearson correlation coefficients.

Lastly, to investigate the relationship between club performance and sponsorship management outcomes, we compiled final club rankings for the 2021 to 2023 spring seasons and examined their associations with subsequent-year sponsorship diversification (Blau index) and continuity (Jaccard similarity). The analysis adopted a lagged structure, where club performance in year x was tested against sponsorship metrics in year *x* + 1, reflecting the causal logic that competitive success may attract or stabilize sponsorship in the following season. Six Spearman correlation tests were conducted to assess monotonic relationships between rank and the Blau/Jaccard indices. This exploratory analysis helps uncover whether performance-driven brand exposure influences managerial sponsorship outcomes.

## 4 Results

### 4.1 Ownership and sponsorship distribution

Table 1 provides a list of ownership structures within the LPL. The LPL is operated under the oversight of Tencent, which also owns the league's intellectual property. Among the 17 clubs, five are owned or controlled by publicly held companies. Six clubs or development zones. Additionally, two clubs, AL and LGD, are led by female CEOs, highlighting a modest representation of gender diversity in club leadership.

**Table 1:** Ownership of league of legends Pro league.

Name	Primary ownership		Note regarding the owner
	Name	Business type	
League of Legends Pro League (LPL)	Tencent	Public	IT conglomerate
Anyone's Legend (AL)	Jiang, Yafei	Private	Managed by Chengdu Star Capital
Bilibili Gaming (BLG)	Bilibili	Public	Media company
EDward Gaming (EDG)	Zhu, Yihang	Private	Backed by a real estate business
FunPlus Phoenix (FPX)	FunPlus	Private	Video game developer and publisher
Invictus Gaming (iG)	Wang, Sicong	Private	Backed by a real estate business
JD Gaming (JDG)	JD.com	Public	E-commerce conglomerate
LGD Gaming (LGD)	Pan, Jie	Private	Backed by its own esports ecosystem
LNG Esports (LNG)	Li-Ning	Public	Sports manufacturing company
Ninjas in Pyjamas (NIP)	He, Youjun	Private^a^	Backed by the NIP Group and a casino business
Oh My God (OMG)	Zhang, Hui	Private	No public information available
Rare Atom (RA)	Hua Xiang Group	Private	Real estate business
Royal Never Give Up (RNG)	Yao, Jincheng	Private	Real estate business
Top Esports (TES)	Topsports	Public	Sports retail business
Thunder Talking (TT)	TTChat	Private	Esports social media platform
Ultra Prime (UP)	Nenking Group	Private	Real estate business
Team WE (WE)	Qujiang New District	Private	Government-sponsored esports industrial base
Weibo Gaming (WBG)	Sina Corporation	Public	Media company

^a^ NIP became a public held corporation in August 2024.

### 4.2 Sponsor composition and volume

**Figure 1:**
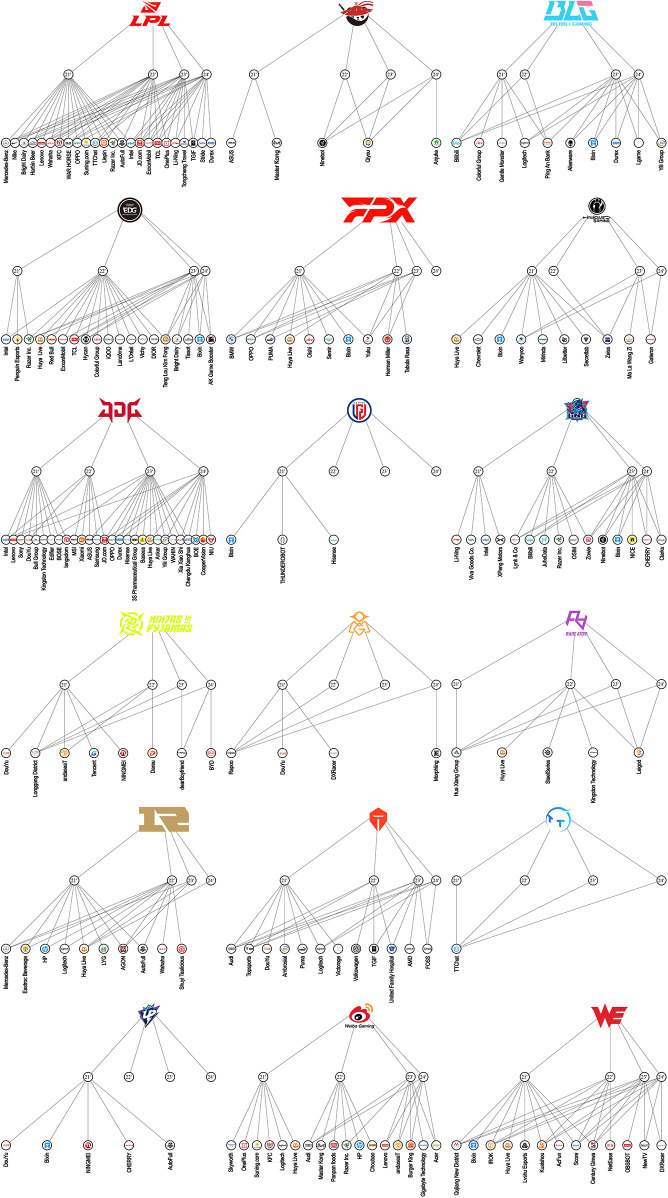
Sponsorship map of league of legends Pro league from 2021 to 2024. Readers are directed to the online version of the high-resolution figure (DOI: 10.6084/m9.figshare.29420846.v1).

As illustrated in Figure 1, the LPL sponsorship network exhibits notable asymmetries in sponsor volume. League-level sponsorship averaged 14 corporate partners per season, whereas clubs averaged 4.5 sponsors. A statistically significant difference in sponsorship count was observed by ownership type: public corporations secured an average of 7.1 sponsors per year, compared to 3.7 among privately held clubs (*p* < 0.05).

### 4.3 Industry profiles of sponsors

Table 2 summarizes league-level sponsors categorized by MSCI industry groups. The food and beverage sector accounted for the largest share of sponsorships, followed closely by technology hardware and equipment—sectors that align naturally with esports' consumer base and digital infrastructure. Other contributing industries included automotive, apparel, and semiconductor sectors.

**Table 2:** Industry group of league-level sponsors.

Industry group	2021		2022		2023		2024		Average	
	Ct.	%	Ct.	%	Ct.	%	Ct.	%	Ct.	%
Automobiles & Components	1	6.7	1	7.1	1	7.1	1	7.7	1	7.1
Commercial & Professional Services	1	6.7	1	7.1	—	—	—	—	0.5	3.6
Consumer Discretionary Distribution & Retail	1	6.7	1	7.1	1	7.1	1	7.7	1	7.1
Consumer Durables & Apparel	1	6.7	1	7.1	2	14.3	2	15.4	1.5	10.7
Energy	—	—	1	7.1	1	7.1	1	7.7	0.75	5.4
Financial Services	—	—	—	—	1	7.1	—	—	0.25	1.8
Food, Beverage & Tobacco	5	33.3	4	28.6	3	21.4	3	23.1	3.75	26.8
Household & Personal Products	—	—	—	—	—	—	1	7.7	0.25	1.8
Media & Entertainment	1	6.7	1	7.1	1	7.1	—	—	0.75	5.4
Semiconductors & Semiconductor Equipment	1	6.7	1	7.1	1	7.1	1	7.7	1	7.1
Technology Hardware & Equipment	4	26.7	3	21.4	3	21.4	3	23.1	3.25	23.2
Total	15	100	14	100	14	100	13	100	14	100

Ct, count.

Table 3 presents the parallel distribution at the club level. Technology hardware and equipment again led in sponsor count, followed by media and entertainment, automotive, and food and beverage. These top sectors, combined with retail and apparel, constituted 74.6% of all club-level sponsors, indicating a relatively narrow commercial footprint compared to traditional sports leagues.

**Table 3:** Industry group of club-level sponsors.

Industry group	2021		2022		2023		2024		Average	
	Ct.	%	Ct.	%	Ct.	%	Ct.	%	Ct.	%
Automobiles & Components	5	5.7	9	12.2	9	10.8	8	12.7	7.75	10.1
Banks	1	1.1	1	1.4	1	1.2	—	—	0.75	1.0
Consumer Discretionary Distribution & Retail	6	6.9	3	4.1	5	6.0	3	4.8	4.25	5.5
Consumer Durables & Apparel	11	12.6	3	4.1	10	12.0	3	4.8	6.75	8.8
Consumer Services	2	2.3	—	—	1	1.2	1	1.6	1	1.3
Equity Real Estate Investment Trusts	2	2.3	2	2.7	2	2.4	2	3.2	2	2.6
Food, Beverage & Tobacco	7	8.0	8	10.8	7	8.4	5	7.9	6.75	8.8
Health Care Equipment & Services	—	—	1	1.4	1	1.2	—	—	0.5	0.7
Household & Personal Products	—	—	4	5.4	3	3.6	5	7.9	3	3.9
Media & Entertainment	23	26.4	10	13.5	12	14.5	11	17.5	14	18.2
Pharmaceuticals, Biotechnology & Life Sciences	—	—	1	1.4	3	3.6	2	3.2	1.5	2.0
Real Estate Management & Development	1	1.1	1	1.4	1	1.2	2	3.2	1.25	1.6
Semiconductors & Semiconductor Equipment	3	3.4	2	2.7	2	2.4	1	1.6	2	2.6
Software & Services	1	1.1	4	5.4	4	4.8	4	6.3	3.25	4.2
Technology Hardware & Equipment	25	28.7	25	33.8	22	26.5	16	25.4	22	28.7
Total	87	100	74	100	83	100	63	100	76.8	100

Ct., count.

### 4.4 Sponsorship continuity

Table 4 reports club-level sponsorship continuity, measured via the Jaccard similarity coefficient. TT emerged as a distinctive outlier, maintaining the exact same sponsors across three consecutive years, though without evidence of responsive strategic adaptation. On average, clubs demonstrated high volatility compared to the league. Ownership status did not significantly influence continuity; the mean volatility index was 0.25 for publicly held clubs and 0.27 for private clubs (*p* = 0.72).

**Table 4:** Sponsorship continuity.

Name	Jaccard similarity coefficient			Volatility index
	21′–22′	22′–23′	23′–24′	
AL	0	1	0.500	0.500
BLG	0.250	0.143	0.778	0.340
EDG	0.077	0.273	0.538	0.231
FPX	0.333	0.500	0	0.255
IG	0.067	0.143	0.333	0.137
JDG	0.125	0.111	0.364	0.142
LGD	0	—	—	—
LNG	0	0.231	0.778	0.400
NIP	0.250	0.200	0.500	0.161
OMG	0.200	1	0.333	0.429
RA	0.111	0.250	1	0.478
RNG	0.636	0.200	0.200	0.252
TES	0.167	0.400	0.400	0.135
TT	1	1	1	0.000
UP	0	—	—	—
WBG	0	0.167	0.556	0.285
WE	0.385	0.455	0.778	0.210
LPL	0.500	0.556	0.556	0.032

### 4.5 Sponsorship diversification

Table 5 displays club-level diversification, as measured by the Blau index. EDG achieved the highest diversification (Blau index = 0.803), while TT again stood out for its complete lack of diversification (Blau = 0). At the league level, the LPL consistently maintained the most diversified sponsorship portfolio. Publicly held clubs demonstrated significantly higher diversification than private clubs (mean Blau index: 0.73 vs. 0.50; *p* = 0.006), reinforcing the stabilizing effect of institutional ownership on commercial partnerships.

**Table 5:** Blau index of sponsorship by industry groups.

Name	2021	2022	2023	2024	Average
AL	0.500	0.500	0.500	0.667	0.542
BLG	0.720	0.500	0.813	0.776	0.702
EDG	0.667	0.847	0.840	0.857	0.803
FPX	0.781	0.778	0.720	—	0.760
IG	0.813	0	0.750	0.500	0.516
JDG	0.716	0.612	0.844	0.847	0.755
LGD	0.667	—	—	—	0.667
LNG	0.750	0.667	0.781	0.735	0.733
NIP	0.720	0.444	0.500	0.667	0.583
OMG	0.444	0	0	0	0.111
RA	0	0.720	0.5	0.500	0.430
RNG	0.656	0.667	0.667	0	0.497
TES	0.816	0.720	0.816	0.750	0.776
TT	0	0	0	0	0
UP	0.480	—	—	—	0.480
WBG	0.722	0.611	0.449	0.560	0.586
WE	0.519	0.778	0.688	0.694	0.700
LPL	0.791	0.827	0.857	0.840	0.829

### 4.6 Managerial modernization

**Figure 2:**
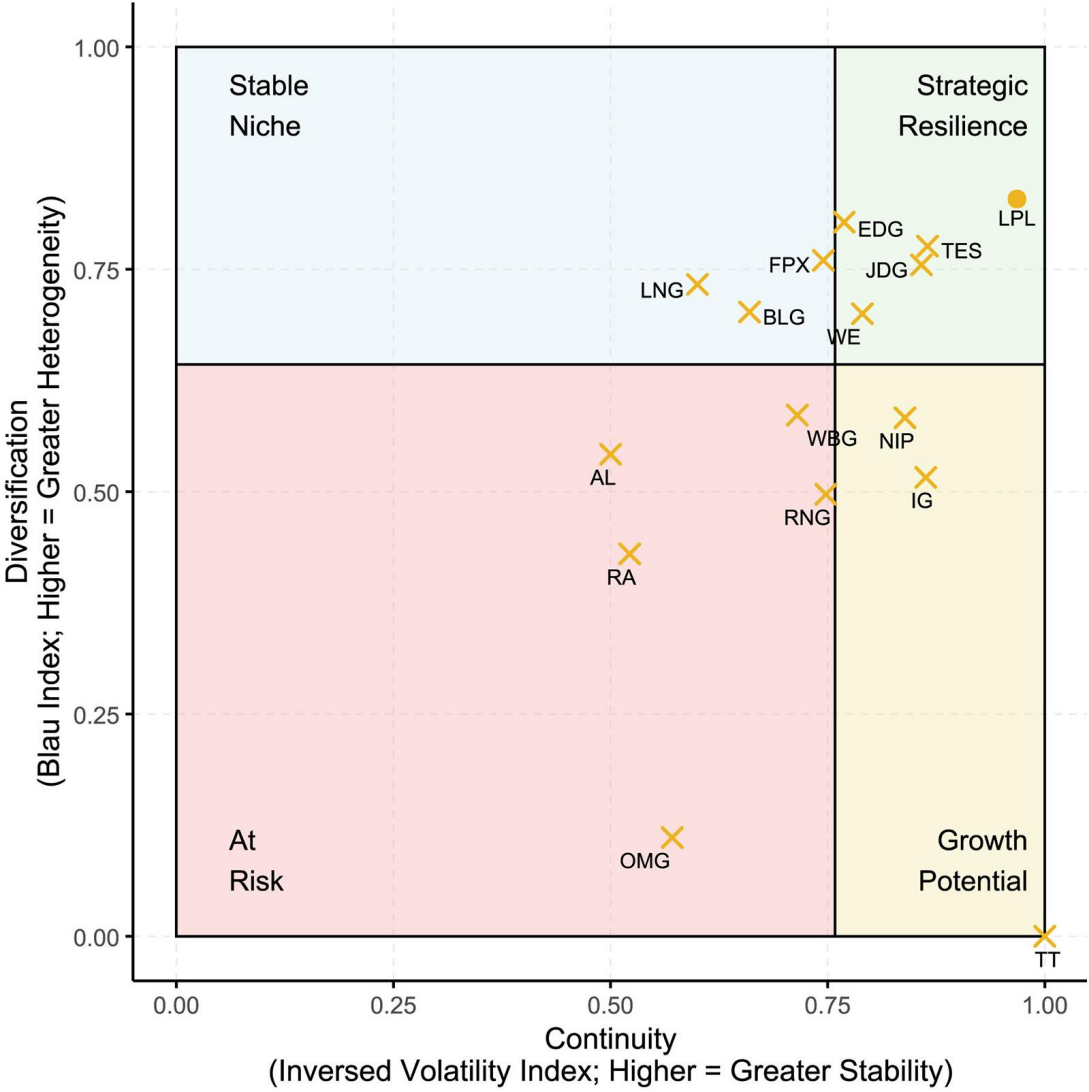
Conceptual matrix of continuity vs. diversification in LPL sponsorship portfolios. The *x*-axis shows the continuity score (one minus volatility index), where higher values indicate greater portfolio continuity. The *y*-axis displays the Blau index, where higher values reflect greater heterogeneity in sponsorship categories. The vertical and horizontal dashed lines indicate median splits used to define four conceptual quadrants: Strategic Resilience (green), Stable Niche (light blue), Growth Potential (yellow), and At Risk (pink). Each “X” denotes a club, while the LPL benchmark is shown as a circle.

Figure 2 cross-plots the continuity score against the Blau index of sponsorship category diversification. The distribution reveals that a small cluster of clubs (e.g., EDG, JDG, TES) occupy the Strategic Resilience quadrant, combining high continuity with diversified sponsor portfolios—a position likely to buffer them from market shocks and sponsor turnover. Several clubs (e.g., LNG, BLG, FPX) fall into the Stable Niche quadrant, showing strong sponsor retention but relatively concentrated portfolios, suggesting limited cross-category reach. Others, such as IG and NIP, display high continuity but low diversification, placing them in Growth Potential, where strategic expansion into new categories could strengthen resilience. Meanwhile, clubs like AL, RA, and OMG appear At Risk, with low continuity and limited diversification, potentially facing greater vulnerability to sponsor loss and revenue volatility. The LPL aggregate portfolio lies at the upper-right edge, reflecting both high continuity and diversification at the league level.

### 4.7 Metric robustness and validity

Robustness checks using alternative metrics affirmed the reliability of the findings. The volatility index based on Jaccard coefficients strongly correlated with the *E* − *I* index (*r* = 0.95). Likewise, the Blau index correlated highly with Shannon's entropy across all four seasons—0.96 (2021), 0.97 (2022), 0.96 (2023), and 0.96 (2024)—demonstrating consistency in the measurement of sponsor diversification.

### 4.8 Performance and sponsorship strategy

Table 6 presents the Spearman correlation results evaluating the link between club performance and subsequent sponsorship metrics. A strong, statistically significant negative correlation was observed between 2021 rank and the Blau index in 2022 (*ρ* = –0.743, *p* = 0.0015), suggesting that higher-performing clubs (i.e., lower ranks) secured more diversified sponsorships in the following year. A similar but marginally significant relationship was found for 2023 rank and the 2024 Blau index (*ρ* = –0.502, *p* = 0.067).

**Table 6:** Spearman correlations between club performance and subsequent-year sponsorship continuity and diversification.

Comparison	Spearman's *ρ*	*p*-value
Rank 2021 vs. Blau 2022	−0.743	0.0015
Rank 2022 vs. Blau 2023	−0.355	0.194
Rank 2023 vs. Blau 2024	−0.502	0.067
Rank 2021 vs. Jaccard 21–22	−0.341	0.180
Rank 2022 vs. Jaccard 22–23	0.557	0.031
Rank 2023 vs. Jaccard 23–24	−0.171	0.543

However, the link between performance and sponsor continuity was less consistent. Most Jaccard-based correlations were statistically insignificant. Notably, one unexpected result emerged: a statistically significant positive correlation between 2022 rank and 2022–2023 sponsor continuity (*ρ* = 0.557, *p* = 0.031), implying that lower-performing clubs were more likely to retain sponsors. This may reflect long-term contractual arrangements or lower performance-related expectations from sponsors.

## 5 Discussion

Through a quantitative analysis of sponsorship portfolios, this study revealed the enigma surrounding continuity and diversification strategies in the LPL. The results demonstrate significant heterogeneity in sponsorship management strategies within the world's most commercial esports league. This finding offers valuable insight into how a rapidly evolving new industry organizes strategic partnerships in the absence of long-established norms.

Sponsorship continuity does not imply a static view of partnership. Tencent, the holder of the LPL brand in China, replaced roughly half of its sponsors every year between 2021 and 2024. Nonetheless, the very low volatility index suggests that Tencent internally employs a mature continuity strategy while reshuffling its sponsorship portfolio. In this way, Tencent effectively “saves three birds with one nest.” First, it strengthens long-term ties with global and domestic industrial giants. For instance, Mercedes-Benz has used its LPL partnership to secure brand prestige among younger Chinese consumers (20), especially in the face of intense competition from domestic EV manufacturers and rising nationalist sentiment (21). Similarly, Bright Dairy, Li-Ning, and JD.com face market saturation and image fatigue, and thus seek brand revitalization through their association with Tencent and LPL. Second, Tencent's approach ensures portfolio sustainability: by maintaining the overall number of sponsors constant, Tencent guarantees a predictable cash flow and preserves the premium value of sponsorship slots within the LPL. Third, by rotating a portion of sponsors annually, Tencent adapts its commercial image to market trends. A prime example is OPPO's aggressive entry into esports, outbidding OnePlus to become the league's smartphone partner. This dynamic restructuring of sponsor portfolios both aligns with real-time shifts in consumer demand and reinforces LPL's reputation as a high-value media platform. Tencent's own approach offers a pathway forward. It demonstrates that continuity and adaptability are not contradictory but complementary. By balancing long-term relationships with selective turnover, Tencent mitigates risk, preserves exclusivity, and ensures that sponsorships remain aligned with evolving market realities.

In contrast, LPL clubs lack a coherent continuity strategy. Most have exhibited a steady decline in sponsors since 2021. The most direct explanation is rooted in financial precarity. Similar to other professional sports clubs in China (22), many esports clubs operate at a loss. To remain viable, they often accept whatever terms sponsors offer, resulting in fragile, short-term arrangements that rarely exceed one year. This behavior is less a deliberate managerial choice than a reactive necessity for survival. The results further reveal that many clubs are either unable or insufficiently equipped to diversify their sponsorship portfolios. Public corporations, by contrast, appear more attuned to the commercial risks of sponsor concentration and proactively manage their brand exposure across industries. This reflects both a stronger corporate governance structure and greater access to marketing resources.

The positioning of clubs within the continuity–diversification matrix further clarifies the variety of commercial strategies at play in the LPL ecosystem. EDG and WE represent two cases of strategic resilience, combining enduring sponsorship ties with a broad cross-category portfolio—a profile consistent with its sustained competitive performance and strong brand equity. EDG, the 2021 League of Legends World Champion, has capitalized on its international visibility, aided by the entrepreneurial background of its owner Edward Zhu, whose extensive business network and managerial experience likely contribute to the club's commercial agility. WE, though less prominent in competitive success, secured substantial public funding from the Xi'an municipal government. This industry-government business incubation has likely strengthened the club's commercial operation capacity and sponsor appeal. Both cases demonstrate how alternative organizational strengths—whether championship success or public institutional support—can substitute for formal ownership advantages in enabling sponsor diversification. In contrast, LNG's placement in the stable niche quadrant reflects a secure but more narrowly focused sponsor base, which may limit brand reach beyond core partner sectors. IG's growth potential status, marked by high continuity but low diversification, suggests that carefully targeted expansion into underrepresented categories could enhance resilience without jeopardizing existing relationships. At the opposite end of the spectrum, AL's at risk positioning underscores how the combination of low continuity and low diversification constrains commercial adaptability, leaving the club more vulnerable to sponsor loss and revenue fluctuations.

However, macrostructural forces have also contributed to the weakening sponsorship climate. The COVID-19 lockdowns in 2020 and 2022 disrupted offline events, forcing a pivot to livestreaming and reducing the effectiveness of jersey branding. Simultaneously, China's digital marketing landscape has shifted dramatically toward algorithm-driven short videos and influencer-centered campaigns (23, 24). Traditional models of esports sponsorship—built on club image, uniform branding, and tournament visibility—have not adapted swiftly enough to compete with these emerging modalities. As such, clubs that fail to evolve face increasing challenges in securing sustainable sponsorship.

Collectively, these findings suggest that the degree of managerial modernization, not merely performance or ownership status, plays a pivotal role in shaping portfolio approach. In esports, where revenue volatility and reputational risks are pronounced, the ability to retain sponsors while cultivating diversification offers clubs a rare dual advantage: financial resilience and brand differentiation. Clubs that lack this managerial capacity are not only more vulnerable to sponsor churn but may also find themselves increasingly marginalized in a maturing and competitive sponsorship ecosystem. This underscores the urgent need for esports clubs to develop internal sponsorship expertise, adopt portfolio-based thinking, and strategically position themselves within broader commercial networks—principles that form the foundation of sustainable sponsorship management.

## 6 Theoretical implications

This study contributes to the growing literature on esports commercialization by advancing our understanding of how sponsorship strategy functions in politically coordinated yet commercially competitive environments. Traditional sports sponsorship, as seen in football, relies heavily on multi-tiered arrangements and legacy branding. For instance, Real Madrid maintains long-standing contracts with Emirates and Adidas while exploring regional marketing partnerships to localize its reach. Esports, however, lacks this hierarchical maturity. Most clubs operate on volatile sponsorships with little room to experiment, let alone secure multi-year deals.

Our findings suggest that blindly adopting the traditional sports sponsorship model may misalign with esports' unique consumption dynamics. For example, jersey visibility—central to traditional sponsorship (25)—carries minimal branding value in esports, as most fan engagement occurs via game screens or livestreaming platforms, not physical arenas. In contrast, if game developers allowed for customizable in-game skins featuring sponsor logos, a new layer of commercial visibility could emerge. This insight opens a novel theoretical direction for future esports marketing research.

Furthermore, our study introduces a methodological contribution. Although the analysis focuses on the LPL, the continuity–diversification framework is portable. Application to other leagues such as LCS or LCK would require harmonizing sponsor category taxonomies, accounting for differences in ownership structure and disclosure norms, and aligning season timing. With these adjustments, the same matrix can position clubs in other contexts and enable cross-league comparison of sponsorship continuity and diversification. We deliberately restrict our empirical scope to the LPL here and view cross-context replication as a next step. These metrics also echo tools used in innovation portfolio analysis, supply chain resilience, and stakeholder mapping—indicating the potential for interdisciplinary extensions.

Uniquely, this study reveals how political economic systems shape managerial intent. In China, ownership of a professional sports club is often less about profit and more about symbolic capital. Corporations, particularly in real estate and retail, may use esports partnerships to gain policy favor or strengthen government relations (26). The Evergrande FC example illustrates how loss-making sports ventures can serve broader business expansion goals (22). This blurs the boundary between financial strategy and strategic influence—a reality that challenges Western economic assumptions embedded in sponsorship theory.

## 7 Managerial implications

Despite the structural uniqueness of the LPL, our findings point to several transferable lessons for esports sponsorship management. First, ownership structure matters: publicly held clubs leveraged stronger governance and marketing resources to achieve greater diversification, suggesting that governance reform or strategic partnerships can enhance portfolio breadth. Second, while competitive success can attract more diverse sponsors, continuity depends on deliberate relationship management rather than seasonal results alone. Third, sustainable portfolios balance long-term anchor sponsors with selective rotation in category-specific partnerships, enabling both stability and adaptability. Finally, diversification across unrelated sectors reduces exposure to reputational or market shocks, strengthening bargaining power with sponsors.

Tencent's league-level strategy exemplifies these principles, maintaining enduring relationships with global brands while adjusting category partners to align with market trends. Smaller clubs, though operating with fewer resources, can adapt this “continuity-with-adaptability” model in scaled form to build resilience under uncertain conditions. Internationally, leagues such as the LCS and LCK can adopt the continuity–diversification framework to anticipate commercial downturns, retool sponsorship architectures, and promote sustainable operations in volatile esports markets.

## 8 Limitations

Several limitations warrant discussion. In this study we did not measure sponsorship revenue, contract value, or renewal fees, and the available data do not permit identification of causal mechanisms behind continuity or change. As a result, we cannot quantify the direct financial impact of continuity and diversification, nor can we determine whether low continuity reflects managerial choices or external shocks. Future work can address these gaps by combining panel data on sponsorship value and renewal rates with qualitative interviews of club commercial directors and sponsors, and by using event-study or difference-in-differences designs around exogenous changes such as policy shifts or macroeconomic downturns. Such mixed-methods designs would allow tests of whether higher continuity and broader diversification translate into higher and more stable sponsorship income, and would help distinguish strategic decisions from external constraints.

In addition, this study focuses solely on the LPL. While it is the world's most developed league in terms of commercial structure, its political-economic context is unique. Chinese clubs may receive indirect subsidies or reputational rewards that insulate them from financial pressures common in market economies. By contrast, clubs in the LCS and LCK typically operate under stricter financial sustainability constraints. Our decision to exclude these leagues was due to structural incompatibility, differences in revenue models, and scope considerations. We encourage future research to apply our framework across regions for comparative analysis.

## 9 Conclusions

This study provides first empirical evidence on how Chinese esports clubs and league manage their sponsorship portfolios. The findings reveal high structural heterogeneity: Tencent, as the league operator, maintains both high continuity and diversification, while many LPL clubs exhibit fragmented, under-diversified, and transient sponsor relationships. These findings have practical and theoretical relevance. They underscore the role of ownership structure, managerial capacity, and brand positioning in determining sponsorship strategy. While public corporations show greater resilience via diversification, private clubs remain vulnerable to market shocks. Meanwhile, performance affects sponsorship outcomes inconsistently, suggesting that relationship-based models—not performance-based sponsorships—may dominate the LPL's future. The study also provides a transferable toolkit for quantifying portfolio approach using established network and diversity indices. These tools allow scholars and practitioners to measure sponsorship sustainability, identify outliers, and benchmark performance against peers across leagues and seasons. As the esports industry navigates increasing economic volatility, ethical scrutiny, and audience fragmentation, this study offers a timely blueprint for sustainable commercial planning.

## Data Availability

The original contributions presented in the study are included in the article/Supplementary Material, further inquiries can be directed to the corresponding author.
